# Improved health outcomes with Etanercept versus usual DMARD therapy in an Asian population with established rheumatoid arthritis

**DOI:** 10.1186/1471-2474-14-13

**Published:** 2013-01-08

**Authors:** Sang-Cheol Bae, Suk Chyn Gun, Chi Chiu Mok, Rezaul Khandker, Henk W Nab, Andrew S Koenig, Bonnie Vlahos, Ron Pedersen, Amitabh Singh

**Affiliations:** 1 Department of Rheumatology, Hanyang University Hospital for Rheumatic Diseases, Seoul 133-792, Korea; 2 Department of Medicine, Hospital Tuanku Ja’afar Seremban, Seremban, Malaysia; 3 Department of Medicine, Tuen Mun Hospital, Hong Kong, Hong Kong; 4 Pfizer Inc., Specialty Care Business Unit, 500 Arcola Road, Collegeville, PA, 19426, USA; 5 Pfizer Inc., Europe, Rome, Italy

**Keywords:** Patient reported outcomes, Etanercept, Rheumatoid arthritis, Asia-Pacific, Health outcomes assessments

## Abstract

**Background:**

Patient reported outcomes (PROs) are especially useful in assessing treatments for rheumatoid arthritis (RA) since they measure dimensions of health-related quality of life that cannot be captured using strictly objective physiological measures. The aim of this study was to compare the effects of combination etanercept and methotrexate (ETN + MTX) versus combination synthetic disease modifying antirheumatic drugs (DMARDs) and methotrexate (DMARD + MTX) on PRO measures among RA patients from the Asia-Pacific region, a population not widely studied to date. Patients with established moderate to severe rheumatoid arthritis who had an inadequate response to methotrexate were studied.

**Methods:**

Patients were randomized to either ETN + MTX (N = 197) or DMARD + MTX (N = 103) in an open-label, active-comparator, multicenter study, with PRO measures designed as prospective secondary endpoints. The Health Assessment Questionnaire (HAQ), Functional Assessment of Chronic Illness Therapy Fatigue Scale (FACIT-Fatigue), Medical Outcomes Short Form-36 Health Survey (SF-36), Hospital Anxiety and Depression Scale (HADS) and the Work Productivity and Activity Impairment Questionnaire: General Health (WPAI:GH) were used.

**Results:**

Significantly greater improvements were noted for the ETN + MTX group at week16 for HAQ mean scores and for proportion of patients achieving HAQ score ≤ 0.5, compared to patients in the DMARD + MTX group. SF-36 Summary Scores for physical and mental components and for 6 of 8 health domains showed significantly greater improvements at week16 for the ETN + MTX group; only scores for physical functioning and role-emotional domains did not differ significantly between the two treatment arms. Greater improvements at week16 were noted for the ETN + MTX group for FACIT-Fatigue, HADS, and WPAI:GH mean scores.

**Conclusion:**

Combination therapy using ETN + MTX demonstrated superior improvements using a comprehensive set of PRO measures, compared to combination therapy with usual standard of care DMARDs plus MTX in patients with established rheumatoid arthritis from the Asia-Pacific region.

**Trial registration:**

clintrials.gov # NCT00422227

## Background

Patient reported outcomes (PROs) are increasingly recognized as a scientifically valid tool for assessing the effectiveness of therapy for patients with rheumatoid arthritis (RA) [1,2]. The noteworthy impact of RA on functional ability of the patient can result in lost productivity, reduced social functioning and overall impaired health-related quality of life (HRQoL) [3]. Treatment options for patients with RA can vary according to disease severity and duration [4,5]. Early initiation with disease modifying anti-rheumatic (DMARD) drugs is now a mainstay of therapy, and the stated goal of therapy is to achieve remission or the lowest possible disease activity state [6,7]. Achieving remission or a lower disease activity state can be challenging, especially in patients with longer duration of RA and more severe disease activity [8]. PROs are especially useful in assessing a given treatment option since they measure dimensions of HRQoL that cannot be captured using strictly objective physiological measures [9].

Treatment options have evolved over the past few decades to include biological DMARDs. Patients who have failed to respond to therapy with synthetic DMARDs may be considered candidates for biological DMARD therapy [4,5]. Previous studies have shown that the fully human anti-tumour necrosis factor (TNF) agent, etanercept (ETN), alone or in combination with MTX, can significantly improve outcomes among patients who have failed to respond to previous therapy with synthetic DMARDs [10-12]. Significantly improved PROs have also been shown in trials comparing combination ETN + MTX with ETN or MTX [13,14].

The APPEAL (Asia-Pacific Study in Patients to be Treated With Etanercept or an Alternative Listed DMARD) trial was conducted to compare the clinical efficacy and safety of combination ETN + MTX with combination DMARD + MTX in patients with moderate to severe rheumatoid arthritis from the Asia-Pacific region [15]. A comparative efficacy trial in this population was important since the normal practice pattern in the region is to start biologics after failure of several DMARDs. Results showed that ETN + MTX in patients with moderate to severe rheumatoid arthritis from the Asia-Pacific region showed superior efficacy compared with usual DMARD + MTX regimens, with similar safety profiles. The objective of the present study was to report the PROs captured in the APPEAL trial.

## Methods

The APPEAL trial was a Phase 4, 16-week, randomized, open-label, active-comparator, parallel-design, outpatient, multicenter study conducted in the Asia-Pacific region, the primary objective of which was to compare efficacy and safety of ETN + MTX with usual DMARD + MTX. The trial was conducted at sites in Hong Kong, India, Malaysia, Philippines, Taiwan, Korea and Thailand between June 2007 and March 2009. Details of the patient population, inclusion and exclusion criteria, and primary and secondary clinical efficacy results are summarized throughout this manuscript and reported in full elsewhere [15]. PROs reported here are prospective secondary endpoints from patients forming the modified intent to treat (mITT) population enrolled in the two treatment groups of the APPEAL study.

Written informed consent was obtained from all patients prior to enrollment. This study was conducted in accordance with the International Conference on Harmonisation Guideline for Good Clinical Practice and the ethical principles that have their origins in the Declaration of Helsinki. Written approval was obtained from Independent Ethics Committees/Institutional Review Boards at each institution participating in the study.

### Patients

The APPEAL study enrolled patients aged 18–69 years at time of consent with active moderate to severe RA, based on 1987 ACR criteria [16] and 28-joint Disease Activity Score [DAS28] ≥3.2, who displayed inadequate response to oral MTX (stable dosing between 7.5 mg/week and 25 mg/week for minimum 3 months) at screening. Patients were randomized to either of two treatment groups in an approximate 2:1 ratio: ETN + MTX (N = 197) or to DMARD + MTX (N = 103). ETN was administered subcutaneously (25 mg per injection) twice weekly. The dose and administration of usual DMARD therapy (defined as the addition of DMARD investigator’s choice to MTX) followed the standard of care and approved local label or recommendations; the three most frequently used DMARDs in the study were leflunomide (n = 69), sulfasalazine (n = 23) and hydroxychloroquine (n = 11). MTX was to be taken orally once weekly as a single dose or in two divided doses on the same day (per local label) and was continued at the same dose throughout the study as at the time of screening (N = 300). Folic acid supplementation was recommended to all patients to minimize side effects associated with MTX.

### Patient reported outcomes instruments

Five instruments were used to evaluate health- and quality of life-related PROs. The Health Assessment Questionnaire (HAQ) assesses functional ability for eight subscales: arising, common daily activities, dressing and grooming, eating, grip, hygiene, reach and walking. The HAQ subscales range from 0 (no difficulty) to 3 (unable to do). The Medical Outcomes Short Form-36 (SF-36) Health Survey assesses health related quality of life in eight domains: bodily pain, general health, physical functioning, role-physical, mental health, role-emotional, social functioning and vitality. SF-36 scores range from 0 (worst) to 100 (best) for each of the eight domains. SF-36 Physical Component Summary scores comprise scores from bodily pain, general health, physical functioning and role physical domains while Mental Component Summary scores are derived from mental health, role-emotional, social functioning and vitality domains.

The Hospital Anxiety and Depression Scale (HADS) is comprised of 7 items each for assessing clinically significant anxiety and depression with possible scores from 0 (best) to 3 (worst) [17]. The Functional Assessment of Chronic Illness Therapy Fatigue (FACIT-Fatigue) Scale Scores range from 0 to 52, with higher scores indicating less fatigue [18]. The Work Productivity and Activity Impairment Questionnaire: General Health (WPAI:GH) instrument measures percentage impairment of usual activities and percentage impairment of work and productivity due to health, with higher scores reflecting higher percentage impairment [19]. Assessments were carried out at baseline, at week 8 and at week 16 (reported here for week 16). Validated translations of instruments, where available, were used in the various countries based on primary language requirements. English versions were used in those countries where feasible.

### Data analysis

Patient reported outcomes data were analyzed from the mITT population, consisting of all patients who received at least one dose of study drug (ETN or DMARD) in combination with MTX and submitted at least one post-baseline assessment. Missing data were imputed using the last observation carried forward (LOCF) method for HAQ scores. Data presented for WPAI:GH scores were analyzed using observed cases only. Analysis of covariance was used to compare changes in continuous PRO variables from baseline between treatment groups (ETN + MTX versus DMARD + MTX). Pair-wise comparisons using ANCOVA were adjusted for baseline scores, country and treatment effects. Response proportions were analyzed using the Cochran-Mantel-Haenszel test, stratified by country.

## Results

A total of 300 patients were enrolled, 197 of whom were randomized to ETN + MTX and 103 to DMARD + MTX. There were 19 discontinuations, 4 (2%) from the ETN + MTX group and 15 (14.6%) from the DMARD + MTX group and thus 281 patients (93.7%) completed the 16-week study [15]. Demographic and baseline disease characteristics were not significantly different between the two treatment groups; patients were entirely of Asian ethnicity, mainly female (91.4% and 88.4% for ETN + MTX and DMARD + MTX groups, respectively) and of mean age (± SD) 48.4 years ± 12.0 and 48.5 years ± 11.3, respectively [15]. Mean disease duration in years (±SD) was 6.5 ± 7.3 and 6.9 ± 8.5, respectively [15]. Mean weekly dosage (range) of MTX was 12.9 mg (1.9-25.0). Prior DMARD use, other than MTX, was reported in 24.4% and 30.1% of patients [15].

Above the improvements on clinical efficacy measures with ETN + MTX compared to DMARD + MTX that were recently reported [15] and that are summarized in the discussion below, patients receiving ETN + MTX reported significantly greater improvements in all five instruments used to assess functional ability through PRO measures compared to patients receiving DMARD + MTX. Mean HAQ scores at baseline were comparable between ETN + MTX and DMARD + MTX groups at 1.37 ± 0.68 and 1.41 ± 0.67, respectively (*P* = 0.592), but by week 16, the mean HAQ score had improved by 49.4% to 0.69 with ETN + MTX whereas the DMARD + MTX group mean HAQ score had improved by 38.3% to 0.87 (*P* = 0.025; Table 1). Using a cut-off value of 0.5 in HAQ scores, which signifies a score representative of the general population, at baseline, 14.2% of ETN + MTX patients and 12.6% of DMARD + MTX patients had mean HAQ scores ≤ 0.5 (*P* = 0.86), whereas at week 16, 51.8% of ETN + MTX patients and 39.0% of DMARD + MTX patients had mean HAQ scores ≤ 0.5 (*P* = 0.048). In a sub-group analysis of patients with ≤ 12 months of disease duration at baseline (not shown in Table 1), 55.8% of ETN + MTX patients at week 16 had mean HAQ scores ≤ 0.5 compared with 41.0% of DMARD + MTX patients (*P* = 0.031).

**Table 1:** Health assessment questionnaire and short form-36 physical and mental component summary scores

**Instrument/**	**ETN + MTX**	**DMARD + MTX**	***P *value**
Time on therapy			
**HAQ Score**	**Mean Score (% Improvement from Baseline) (N)**		
Baseline	1.37 (0%) (N = 197)	1.41 (0%) (N = 103)	
Week 16	0.69 (49.4%) (N = 193)	0.87 (38.3%) (N = 100)	0.025*
**HAQ Proportion**	**Patients achieving HAQ Score ≤ 0.5**, **Proportion (%)**		
Baseline	28/197 (14.2%)	13/103 (12.6%)	
Week 16	100/193 (51.8%)	39/100 (39.0%)	0.048**
**SF**-**36 Physical Component Summary Scores**	**Mean Score (% Improvement from Baseline) (N)**		
Baseline	30.5 (0%) (N = 193)	30.1 (0%) (N = 100)	
Week 16	40.4 (31.4%) (N = 189)	37.3 (22.8%) (N = 96)	0.003*
**SF**-**36 Mental Component Summary Scores**	**Mean Score (% Improvement from Baseline) (N)**		
Baseline	42.9 (0%) (N = 193)	42.4 (0%) (N = 100)	
Week 16	50.2 (17.5%) (N = 189)	47.8 (13.3%) (N = 96)	0.047*

Scores at Baseline and Following 16 Weeks of Therapy in Asia-Pacific Rheumatoid Arthritis Patients Treated With ETN + MTX versus DMARD + MTX (Last Observation Carried Forward Analysis).

DMARD: Disease Modifying Antirheumatic Drug; ETN: etanercept; FACIT-Fatigue: Functional Assessment of Chronic Illness Therapy- Fatigue; HAQ: Health Assessment Questionnaire; MTX: methotrexate; SF-36: Medical Outcomes Short Form-36 Health Survey.

Values have been rounded.

*ANCOVA, considering the factors ‘baseline score + therapy + country’.

**Cochran-Mantel-Haenszel Test.

**Figure 1:**
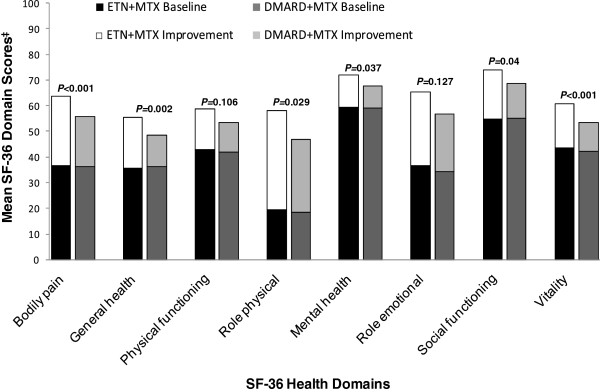
**Short Form**-**36 Individual Domain Scores.** Scores at Baseline and Improvement Following 16 weeks of Therapy with ETN + MTX or DMARD + MTX (Last Observation Carried Forward Analysis)

Summary measures of physical and mental health items from the SF-36 instrument are also presented in Table 1. Mental and physical component summary scores were both similar between the two groups at baseline. At week 16, physical component summary scores had improved by 10.0 points (31.4%) and 7.1 points (22.8%) in the ETN + MTX and DMARD + MTX groups, respectively, while mental component summary scores were improved by 7.3 points (17.5%) and 5.4 points (13.3%), respectively. The improvements were significantly greater among the ETN + MTX patients compared to DMARD + MTX patients for both physical (*P* = 0.003) and mental (*P* = 0.047) component summaries. In terms of individual SF-36 domains, mean SF-36 scores were significantly higher for the ETN + MTX group compared to the DMARD + MTX group for six of eight domains; the two domains where improvements were similar between the two treatment groups were physical functioning and role limitations-emotional (Figure 1). Within the ETN + MTX group, the greatest percentage improvements from baseline were observed for: role limitations-physical (195%), role limitations-emotional (82%), bodily pain (72%) and general health (55%) (Figure 1).

Mean and percentage improvements in the HADS are shown in Table 2. Statistically significant greater improvements were observed in the ETN + MTX patient group compared to the DMARD + MTX group in Anxiety subscale scores—29.1% improvement versus 18.5% improvement, respectively (*P* = 0.026) as well as Depression subscale scores—28.7% improvement versus 16.4% improvement, respectively (*P* = 0.016). Both groups reported similar fatigue rating scores at baseline using the FACIT-Fatigue instrument, however the mean percentage improvement in the FACIT-Fatigue scale was significantly greater in the ETN + MTX group compared with the DMARD group at week 16 (28.0% versus 10.4%, *P* = 0.001, Table 2).

**Table 2:** Hospital anxiety and depression scale scores and functional assessment of chronic illness therapy-fatigue scores

**Instrument/**	**ETN + MTX**	**DMARD + MTX**	*P *Value*
Time on therapy	**Mean Score (% Improvement from Baseline) (N)**		
**Anxiety Subscale**			
Baseline	8.27 (0%) (N = 196)	8.19 (0%) (N = 103)	
Week 16	5.84 (29.1%) (N = 192)	6.69 (18.5%) (N = 100)	0.026
**Depression Subscale**			
Baseline	7.62 (0%) (N = 196)	7.85 (0%) (N = 103)	
Week 16	5.42 (28.7%) (N = 192)	6.56 (16.4%) (N = 100)	0.016
**FACIT**-**Fatigue**			
Baseline	28.1 (0%) (N = 146)	30.1 (0%) (N = 76)	
Week 16	36.2 (28.0%) (N = 143)	33.2 (10.4%) (N = 73)	0.001

Scores at Baseline and Following 16 Weeks of Therapy in Asia-Pacific Rheumatoid Arthritis Patients Treated With ETN + MTX versus DMARD + MTX (Last Observation Carried Forward Analysis).

DMARD: Disease Modifying Antirheumatic Drug; ETN: etanercept; FACIT-Fatigue: Functional Assessment of Chronic Illness Therapy- Fatigue; MTX: methotrexate.

*ANCOVA, considering the factors ‘baseline score + therapy + country’.

The Work Productivity and Activity Impairment Questionnaire was used to quantitatively assess percentage impairment of usual activities among all patients, as well as work- and productivity-related effects of RA on that portion of both groups who were employed (Table 3). Both groups reported similar percent usual activity impairment (approximately 59%) due to health at baseline (Table 3; *P* = 0.983). At week 16, percent usual activity impairment due to health declined to 41% in the DMARD + MTX group and to 30% in the ETN + MTX group, a significant difference between the two groups based on all patients (*P* < 0.001). Employed patients comprised approximately 30% of patients from both groups who reported outcomes at week 16. In this sub-group, significant improvements were noted at week 16 for all three indicators of work productivity. Notably, overall mean work impairment due to health problems was reduced from 52.3% to 25.1% in the ETN + MTX group whereas the reduction in the DMARD + MTX group was smaller, from 56.3% to 41.6% (*P* = 0.004, Table 3). Work time missed due to health was reduced by 80.7% in the ETN + MTX group at week 16, while the DMARD + MTX group reported an average reduction of missed work time of 21.7% at week 16 (*P* = 0.002, Table 3).

**Table 3:** Work productivity and activity impairment questionnaire

**Instrument Subscale**/	**ETN + MTX**	**DMARD + MTX**	*P *value*
Time on Therapy	**Mean Score (% Improvement from Baseline) (N)**		
**Percent activity impairment due to health**			
Baseline	58.6 (0%) (N = 197)	58.6 (0%) (N = 103)	
Week 16	30.0 (48.7%) (N = 186)	41.0 (29.3%) (N = 83)	< 0.001
**Percent impairment while working due to health problem**			
Baseline	48.9 (0%) (N = 62)	51.4 (0%) (N = 37)	
Week 16	24.1 (49.6%) (N = 54)	37.3 (23.6%) (N = 26)	0.012
**Percent overall work impairment due to health problem**			
Baseline	52.3 (0%) (N = 61)	56.3 (0%) (N = 37)	
Week 16	25.1 (51.0%) (N = 53)	41.6 (22.0%) (N = 26)	0.004
**Percent work time missed due to health**			
Baseline	10.6 (0%) (N = 63)	14.9 (0%) (N = 37)	
Week 16	2.04 (80.7%) (N = 55)	10.7 (21.7%) (N = 26)	0.002

General Health at Baseline and Following 16 Weeks of Therapy in Asia-Pacific Rheumatoid Arthritis Patients Treated With ETN + MTX versus DMARD + MTX (Observed Case Analysis).

DMARD: Disease Modifying Antirheumatic Drug; ETN: etanercept; MTX: methotrexate.

*ANCOVA, considering the factors ‘baseline score + therapy + country’.

## Discussion

The APPEAL trial compared efficacy, safety and PROs with ETN + MTX versus DMARD + MTX in patients from Asia-Pacific countries with moderate to severe RA, a mean disease duration of 6.7 years, and sub-optimal response to MTX monotherapy for a minimum of three months. Detailed results of primary clinical endpoints for ETN + MTX versus DMARD + MTX groups, reported by Kim and colleagues[15], showed significantly greater ACR response area under the curve, proportion of subjects achieving ACR 20, 50 and 70 responses at Week 16, numbers achieving low disease activity scores (DAS28 < 3.2), and improvements in DAS28 score, pain visual analogue scale, the health assessment questionnaire, and physician and patient global assessments. Results in the same subjects on health outcomes evaluations, presented here, demonstrate that in addition to improvements in objective clinical measures of disease activity, combination therapy with ETN + MTX provided statistically significant and clinically meaningful improvements in PROs compared to combination therapy using MTX and usual DMARDs.

The mean HAQ scores at baseline were not significantly different between the two groups and both groups of patients reported significant improvements. Slightly more than half (52%) of patients on ETN + MTX achieved a HAQ score lower than 0.5 at week 16, which is representative of the general population, compared to 39% of patients on DMARD + MTX. Furthermore, the ETN + MTX group improved their mean HAQ scores by 49% compared to 38% for the DMARD + MTX group. Two other trials reporting on HAQ scores with MTX/ETN combination therapy have reported percentage improvements in HAQ scores of approximately 50-60% over a period of 52 weeks [13,14]. By way of comparison, Kosinski *et al*. concluded from a study of rheumatoid arthritis patients that an average percentage improvement of 27% in HAQ scores was clinically significant and could be correlated with meaningful changes in other disease severity indicators such as joint swelling and tenderness counts, patient and physician global assessments, and pain [9].

Rheumatoid arthritis patients generally report higher levels of severe fatigue that can exacerbate other symptoms of the disease, worsen physical and emotional well-being and interfere with employment, social and family opportunities and obligations [20,21]. Fatigue is a common and debilitating symptom in RA patients and both the American College of Rheumatology and European League Against Rheumatism have highlighted the need for more information on fatigue from clinical trials, using validated fatigue scales [22]. Both arms in this study reported a lessening of fatigue symptoms as measured using the FACIT-Fatigue instrument, however, the ETN + MTX arm showed a significantly larger reduction of fatigue symptoms after 16 weeks (8 points or 28% improvement) compared to the DMARD + MTX arm (3 points or 10.4% improvement). The clinical significance of this reduction can be understood through a comparison with other studies that have assessed the magnitude of changes in the FACIT-Fatigue scale that are associated with minimal clinically important differences in RA patients. Cella *et al*. [18] estimated a distribution-based minimally important difference in FACIT-Fatigue scores of 4.1, whereas Pouchot *et al*. [23] estimated a regression-based minimal clinically important difference of 8.3 points on the FACIT-Fatigue 52 point scale, which was used in our study.

The mean physical and mental health component summary scores derived from the SF-36 were not different between the two arms at baseline. However, after 16 weeks, both scores improved by a higher percentage for patients randomized to ETN + MTX compared to those on DMARD + MTX. Improvement in absolute score for the ETN + MTX group was higher by a magnitude of 2.7 points for the physical component summary, which is within the range of an accepted minimally clinically important difference (MCID) of 2.5-5 [24]. The mean for mental component score, however, did not meet that threshold. Improvement for the ETN + MTX group was higher than the DMARD + MTX group for six of the eight domain scores by a magnitude within a range of what is considered a MCID for individual domains (5–10 points) [25]. The two domains that did not meet the MCID threshold are mental health and physical functioning.

Not surprisingly, anxiety and depression coexist with RA and tend to be positively correlated with disease severity and duration [26]. A previous study of patients with early RA indicated that anxiety and depression were improved, along with other PRO measures, with ETN + MTX and that these were associated with achieving clinical remission [13]. The significantly greater improvements in the HADS measures for patients on ETN + MTX thus reflect the consistently more positive outcomes in this subject group seen in the other PRO measures. These improvements in functional ability and psychological well-being were further confirmed through a positive impact on work productivity and activity as measured by WPAI:GH. The percentage activity impairment due to health in all patients, as assessed by the WPAI:GH instrument, was improved more substantially in the ETN + MTX group compared to the DMARD + MTX group, by 49% compared to 29%, respectively. In a subgroup of patients who were employed upon enrollment, the WPAI:GH instrument showed an 81% improvement in missed time from work due to health in the ETN + MTX group, compared to a 22% improvement in missed time in DMARD+MTX arm.

There are limitations to this study and the analysis of PRO data. Since the APPEAL trial used an open-label study design, these PRO results may be considered less rigorous than a blinded study. However, this design allowed comparison of combination therapy approaches that incorporated the usual standard of care (synthetic DMARDs) versus ETN in routine clinical settings. The short-term follow-up period (16 weeks) may also be considered a limitation; however this is longer than the 3-month period recommended by EULAR for assessment of current treatment failure or success for RA. Thus, results from the 16 week follow-up period are valid in informing clinical decision making. Further, this study included patients who had not improved while on methotrexate for at least three months, thus the results may not be generally applicable to, for example, DMARD naïve patients. It is however interesting that similar improvements in HAQ and SF-36 scores were demonstrated over 52 weeks in patients from the COMET trial with early RA (mean disease duration of 9 months) [13]. Lastly, the subject population comes from a limited number of countries in this region and may not be generalizable to the entire region or even to all countries studied due to the limited sample size.

## Conclusions

The APPEAL trial provides an evidence base that addresses two gaps in the published literature concerning TNF inhibitor therapy for treatment of RA. First, there is a lack of data on TNF inhibitor therapy from clinical trials originating from the Asia-Pacific region, and second, there is a recognized lack of head-to-head trials comparing outcomes from combination therapies involving synthetic versus biologic DMARDs [27]. This study extends the findings regarding the impact of combination therapy using ETN and MTX on a comprehensive set of PRO measures in patients from the Asia-Pacific region with established rheumatoid arthritis, and reinforces the efficacy of ETN + MTX in these patients.

## Abbreviations

(ETN): Etanercept; (MTX): Methotrexate; (DMARDs): Disease modifying antirheumatic drugs; (PRO): Patient reported outcome; (RA): Rheumatoid arthritis; (HAQ): Health Assessment Questionnaire; (FACIT-Fatigue): Functional Assessment of Chronic Illness Therapy Fatigue Scale; (SF-36): Medical Outcomes Short Form-36 Health Survey; (HADS): Hospital Anxiety and Depression Scale; (WPAI:GH): Work Productivity and Activity Impairment Questionnaire: General Health; (HRQoL): Health-related quality of life; (TNF): Tumour necrosis factor; (mITT): Modified intent to treat; (LOCF): Last observation carried forward; (ACR-N-AUC): American College of Rheumatology response area under the curve; (MCID): Minimally clinically important difference.

## Competing interests

At the time of the study, authors Khandker, Nab, Koenig, Vlahos, Pedersen, and Singh were employed by, and the work was sponsored by, Pfizer, Inc. Authors Bar, Gun and Mok declare that they have no competing interests.

## Authors’ contributions

All authors participated in the design of the study. RK, AK, BV, AS, HN and RP participated in the statistical analysis. All authors contributed to the draft manuscript, and read and approved the final manuscript.

## Pre-publication history

The pre-publication history for this paper can be accessed here:

http://www.biomedcentral.com/1471-2474/14/13/prepub
